# Feeling Unsafe at School and Associated Mental Health Difficulties among Children and Adolescents: A Systematic Review

**DOI:** 10.3390/children8030232

**Published:** 2021-03-17

**Authors:** Yuko Mori, Elina Tiiri, Prakash Khanal, Jayden Khakurel, Kaisa Mishina, Andre Sourander

**Affiliations:** 1 Department of Child Psychiatry, University of Turku, 20014 Turun Yliopisto, Finland; prakash.khanal@utu.fi (P.K.); jayden.khakurel@utu.fi (J.K.); kaisa.mishina@utu.fi (K.M.); 2 INVEST Research Flagship Center, University of Turku, 20014 Turun Yliopisto, Finland; elina.tiiri@utu.fi (E.T.); andsou@utu.fi (A.S.); 3 Department of Child Psychiatry, University of Turku and Turku University Hospital, 20521 Turku, Finland; 4 Department of Nursing Science, University of Turku, 20014 Turun Yliopisto, Finland

**Keywords:** school safety, school climate, feeling unsafe, mental health, systematic review, adolescents, children, bullying, victimization

## Abstract

This study systematically reviewed the literature on perceived school safety. We investigated the prevalence, factors and associated mental health difficulties, as well as cross-cultural findings. Five databases were searched up to 9 February 2021 for peer-reviewed papers published in English. We included quantitative studies that explored the perception of school safety among children and adolescents. The reference lists of the selected papers were also searched. We conducted a narrative synthesis of the included studies. The review included 43 papers. The mean prevalence of the students who felt unsafe at school was 19.4% and ranged from 6.1% to 69.1%. Their perceived safety was associated with a wide range of personal, school, and social factors. Not feeling safe at school was related to being victimized and mental health difficulties, including depressive symptoms and suicidal behavior. Higher perceived school safety was associated with measures such as the presence of a security officer and fair school rule enforcement. The results showed the lack of cross-cultural studies on perceived school safety. Empirical studies are needed that examine the mechanisms of school safety, using valid measures. A clear definition of school safety should be considered a key aspect of future studies.

## 1. Introduction

Researchers have increasingly focused on school safety in the last 20 years and there has been a greater emphasis on physical threats such as gun-related school violence which has received extensive media coverage [1,2,3,4,5]. Another well-known global threat to students’ safety is bullying which takes different forms, namely physical, verbal, relational, and damage to property [6]. School safety is defined by the United Nations Educational, Scientific and Cultural Organization as the process of establishing, and maintaining, a school that is a physically, cognitively, and emotionally safe space for students and staff to carry out learning activities [7]. The United Nations’ Sustainable Development Goals also state that schools should provide safe, non-violent, inclusive, and effective learning environments for all [8].

School safety needs to be examined in terms of both physical and psychological safety. Just because a school is free from violence, it does not necessarily mean it provides a safe environment [2]. Students may worry about their safety even though they do not face any danger from violence or bullying [9]. Safe schools are those where students do not experience fear or anxiety of any danger that can weaken their cognitive ability and restrict the learning process [2]. Students need psychological safety to learn effectively [10].

School safety is often included in studies as one of the aspects of school climate. However, in previous literature, the operationalization of school climate is diverse [11] and many studies do not include school safety as a dimension of school climate [12,13,14,15]. Furthermore, school safety has been conceptualized as an independent outcome of a positive school climate in research [16]. School climate has been reported to be associated with mental health difficulties and bullying victimization [17,18]. However, it is not clear whether perceived school safety itself accounts for these associations or if they can be explained by other aspects of school climate. In this systematic literature review, we focus on perceived school safety.

Current literature on school safety is scattered, and it is necessary to get an overview on what the scholars have discussed on the topic and identify the knowledge gap. There have not been any systematic reviews of the literature on perceived school safety providing a comprehensive overview of studies on how safe children and adolescents felt at school. The aim of this study was to conduct a systematic review on the topic and describe the existing literature and fill that knowledge gap. To do that, we investigated the prevalence of students who felt unsafe at school and examined the factors that were associated with perceived school safety, with a particular focus on any association with mental health difficulties. Bronfenbrenner’s Ecological Systems Theory was used to organize the findings on factors associated with perceived school safety [19,20]. We also wanted to shed light on the findings of cross-cultural studies on school safety, as researchers have suggested that it is vital to use a cross-cultural perspective to explain cultural variances in perceived school safety [21,22]. The findings of this study will directly help educators, researchers, and policy makers to make decisions based on evidence-based knowledge to strengthen the safety of schools.

## 2. Materials and Methods

This systematic review was conducted using the Preferred Reporting Items for Systematic Reviews and Meta-Analyses (PRISMA) guidelines [23,24,25] and the Synthesis without meta-analysis guidelines [26]. Our systematic literature review protocol was developed and registered in PROSPERO on 1 March 2020 and was last updated on 17 August 2020 (registration number CRD42020171435).

### 2.1. Search Strategy

A comprehensive literature search was conducted on PROSPERO to ensure there were no existing systematic literature reviews or on-going reviews of school safety among children and adolescents. The research questions for the review were developed based on the population, intervention, control and outcomes criteria approach [25]. The study population was children and adolescents enrolled at school, the intervention was the schools, and the outcome was the students’ perceived school safety. The focus of this study was to explore the perceptions of students’ safety at school, not to compare it with others who were not at school. Therefore, a comparison group was not applicable to this study.

The literature search was conducted from 30 January to 5 February 2020 and was last updated on 9 February 2021 to find recently published papers. Five electronic databases were chosen because of their relevance to the field of mental health and education: PubMed, Web of Science, ERIC, PsycINFO, and CINAHL. The search strings used for each database are provided in Supplementary Table S1. Additional literature was identified by assessing the reference lists of the selected papers to ensure that all the potentially eligible studies were included [27].

### 2.2. Study Selection

**Figure 1:**
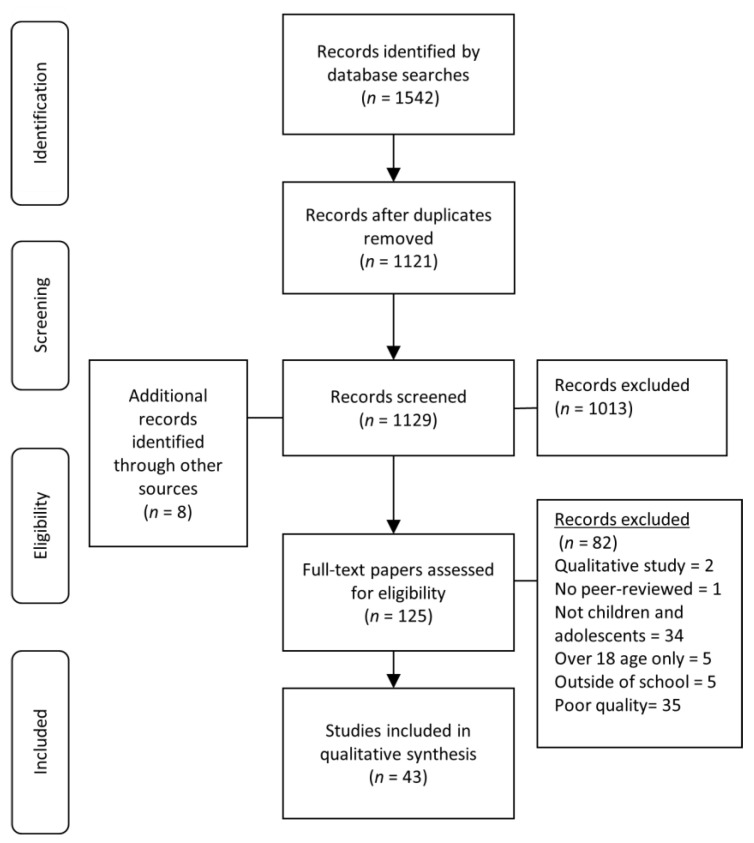
Flow chart used to select papers related to perceived school safety in the present review.

Two authors (YM and PK) independently scanned the titles and abstracts of the papers retrieved from the searches and the papers that did not have an abstract or a full text version were excluded. All searched records were inputted to RefWorks (Ex Libris Ltd., Jerusalem, Israel), and the decisions made during the selection process are reported in the PRISMA flow diagram (Figure 1).

The search was limited to peer-reviewed papers published in English in scientific journals. Case reports, conference abstracts, book chapters, trial registers, Internet resources, and unpublished records were excluded. There were no restrictions on the country of origin. We included quantitative studies that examined the general population of children and adolescents who were enrolled at school. Children and adolescents in school settings, elementary school to high school, were included, and data from individuals only over 18 years old such as college/university students were excluded. Studies that included teachers and parents were only included if the results were reported separately for children and adolescents. Studies on safety outside schools, such as at home and in the community, were excluded.

The main outcome of this review was how safe students felt at school, and this meant that we needed to be clear about how we defined the word safe. The studies that we included used various questions to measure school safety, such as asking students whether they felt fearful or secure at school. Studies that used various synonyms for safe were included in this review. School safety is often included in studies that discuss it as one of the aspects of the school climate. These were only included if the results for school safety were reported separately.

### 2.3. Full Texts and Quality Assessment

The search identified 1129 papers and two authors (YM and PK) carried out screening on the papers based on their titles and abstracts. Any disagreements were resolved between the two authors (YM and PK), with advice from a third author if necessary (ET). Finally, the two authors (YM and PK) assessed the full texts of the 125 papers and identified 43 papers met the eligibility criteria. YM and PK evaluated the quality of the evidence, and any risk of bias in reporting the findings, by using the Quality Assessment Tool for Observational Cohort and Cross-Sectional Studies [28]. The quality of each study was rated as good, fair, or poor. Studies that were rated poor were excluded from this review, to ensure the quality of evidence.

### 2.4. Data Extraction and Data Synthesis

The data extraction was conducted by YM. Excel version 2010 for Windows (Microsoft Corp, Redmond, WA, USA) was used to manage the data and PK checked and verified the accuracy and completeness of the data extraction. The extracted data included the name of the first author, publication year, country, participants, response rate, age range, measures, responses, the year the data was collected, research design, setting, the estimated prevalence of school safety, associated factors, and associated mental health difficulties. Any disagreements in the data extraction were resolved through discussion or consultation between the two reviewers (YM and PK), and a third author helped resolve any disagreements (ET).

The inter-rater reliability of the assessment process was assessed using Cohen’s kappa, and the scores were 0.90 (almost perfect agreement) for the study selection, 0.62 (substantial agreement) for the quality assessment, and 1.00 (perfect agreement) for the data extraction.

Our aim was to present a narrative synthesis of the findings from the included studies, not to conduct a meta-analysis [29]. To ensure consistency across the results, we decided beforehand how the results from each study would be reported [26]. When the prevalence has been reported using a four-point Likert scale, that is divided into feeling safe and feeling unsafe. For instance, feeling safe combines agree and strongly agree and feeling unsafe combines disagree and strongly disagree. Studies that used a five-point Likert scale had an inconsistent approach to presenting prevalence, and we divided the scale responses into feeling safe and feeling unsafe as presented in these studies. Associated factors were organized according to the environment: the child’s immediate environment, interrelations between the different environments that surround them, and environments that the child does not actively participate in [20]. It also includes cultural contexts, life transitions, and sociohistorical events.

## 3. Results

### 3.1. Study Characteristics

We found that 43 papers met the inclusion criteria and these 40 cross-sectional studies, and three longitudinal study underwent data extraction. Of these, 17 papers addressed the prevalence of school safety, 42 papers presented factors associated with perceived school safety, and 10 discussed mental health difficulties related to school safety. Only one cross-cultural study met our criteria and was included.

We reviewed 34 studies from North America: 32 from the United States and two from Canada. One study was from South America, Chile. There were also four studies from Europe, two from the Netherlands, one from Italy and one from Finland. The final three were from Asia, two from Israel, and one from Japan. The response rates ranged from 50–100% in the 33 studies that reported the rates. The number of participants ranged from 542 to 159,630, and they were in the 3rd to the 12th grade. There was one study just from elementary school settings and 38 studies just from secondary school. Four studies covered both elementary and secondary schools. Although we did not set any time limits for the publications, the relevant studies started to appear around 2004, and 30 of the 43 papers were published between 2016–2020.

All the studies used self-report measures to assess how safe the children and adolescents felt at school. A single item was used to measure their feelings in 25 studies, for example, whether they agreed or disagreed with the statement “I feel safe at school”. The other 18 studies used multiple items, such as how safe they felt in the classrooms, corridors and toilets. Most studies used 4- or 5-point Likert scale. The major limitation was that the items that assessed perceived school safety did not define school safety or what it meant to feel safe at school. Table 1 summarizes the studies that were included, and Table S2 provides further details of the included studies.

The quality assessment rated 23 studies as good and 20 as fair. The most common risk of bias was selection bias, followed by no clear description of the research questions and measurement bias. Another common limitation was inadequate adjustments for potential confounders (Table S3).

### 3.2. Prevalence of Perceived School Safety

Table 2 provides a summary of findings from cross-sectional studies which provided the prevalence of school safety. The mean prevalence of students who felt unsafe at school was 19.4% (range 6.1–69.1%). Three studies presented separate data by sex [30,31,32]. The mean prevalence for boys was 31.5% (11.5–68.4%), and for girls, it was 30.7% (10.4–69.9%). Two studies reported the prevalence separately for sexual minority youths (28.8, 39.7%) and non-sexual minority youths (17.2, 20.1%) [22,33].

**Table 2:** Summary of findings from cross-sectional studies which provided the prevalence of school safety.

Studies	Participants, *n*	Response	Design	School	Unsafe Total	Unsafe Boys	Unsafe Girls	Safe Total	Safe Boys	Safe Girls	Certainty of Evidence
[34]	11,986	4-point Likert	CS	S	9.8			90.2			Fair—risk of measurement bias
[31]	20,138	Yes/No	CS	E/S	11.0	12.1	10.4	NR			Fair—risk of measurement bias
[35]	5138	5-point Likert	CS	S	10.2			89.8			Fair—risk of measurement bias
[36]	122,840	4-point Likert	CS	S	18.1			81.9			Fair—risk of selection bias
[37]	75,590	4-point Likert	CS	S	6.1			93.9			Good
[32]	1865	4-point Likert	CS	S	69.1	68.4	69.9	30.8	31.6	30.1	Fair due to risk of measurement bias
[38]	1249	4-point Likert	CS	S	30.9			69,1			Fair due to risk of confounding bias
[39]	4118	4-point Likert	CS	S	25.0			75.0			Fair—risk of confounding bias
[40]	658,122	4-point Likert	LS	S	15.1			84.9			Good
[41]	7958	4-point Likert	CS	S	22.1			77.9			Fair—risk of measurement bias
[42]	71,560	Yes/No	CS	S	15.7			84.3			Fair—risk of selection bias
[33]	542	Yes/No	CS	S	26.9			73.1			Fair—study design
[43]	126,868	4-point Likert	CS	S	7.6			92.4			Good
[22]	9619	Yes/No	CS	S	18.9			81.1			Good
[44]	2231	5-point Likert	CS	S	NR			58.0			Fair—risk of selection bias
[30]	3997	4-point Likert	CS	S	2008: 13.1	14.7	11.0	86.9	85.3	89.0	Good
					2014: 11.4	11.5	11.2	88.6	88.5	88.8	

Note: Schools: elementary (E), secondary (S), CS = Cross-sectional, LS = Longitudinal, NR = Not reported. The numbers have been rounded to one decimal point.

### 3.3. Factors Associated with Perceived School Safety

There were 18 studies that compared perceived school safety separately among sexes. Six studies found that boys were more likely to perceive their schools unsafe than girls [31,34,38,45,46,47], and five studies found that girls reported lower perceptions of school safety than boys [39,42,48,49,50]. Sexual minority youths were less likely to feel safe at school than non-sexual minority youths [22,33,34,51]. Most studies found that non-white respondents were less likely to report feeling safe at school compared to white respondents [34,35,39,43,45,48,49,50]. Four studies reported that older students were more likely to report feeling unsafe at school [38,49,52,53] while three studies reported the opposite [42,47,48]. Two studies reported that students from lower socioeconomic status backgrounds were more likely to perceive their school as unsafe [38,49].

Ten studies reported a significant association between mental health difficulties and a sense of safety at school. Depression and suicidal behavior were the most frequently reported issues. Studies consistently reported that feeling unsafe at school was related to depressive symptoms [45,54,55,56]. Feeling safer at school tended to decrease the probability of suicide attempts and suicidal ideation [55,56,57,58,59]. Self-harming behavior was also found to be associated with a sense of safety at school [32,59]. Yablon [60] found that feeling safe at school had significant associations with fewer post-traumatic stress disorder symptoms and less post-traumatic growth, which means that positive changes resulted from traumatic life events. Nijs et al. [61] used the self-reported Strengths and Difficulties Questionnaire to examine the association between mental health problems and perceived school safety. They found a strong association with the three subscales that measured emotional problems, peer problem, and conduct problems. However, there was no significant association with hyperactivity symptoms and prosocial skills.

#### 3.3.1. Immediate Environment

Victimization was associated with negative perceived school safety in 20 studies. The most common type of victimization was bullying, followed by youth violence, emotional or psychological violence, and witnessing violence. Three studies showed that those who bullied others were more likely to feel unsafe at school than those who stood by and witnessed bullying [30]. Moreover, Bachman et al. [31] found that witnessing bullying was significantly associated with low perceived school safety for 5th-grade students and 8th-grade girls, but not 8th-grade boys. Esselmont [62] found that the direct effects of victimization on perceived school safety were stronger for boys than girls. Most of the studies that have investigated the association between bullying victimization and school safety have only focused on traditional bullying and only two studies covered cyberbullying [53,63].

Several school-related factors were associated with perceived school safety. Five studies found a consistent negative association between feeling unsafe at school and educational attainment [40,42,50,55,64]. A large scale longitudinal study [40] found that future reported perceived school safety does not affect educational attainment in the previous year, suggesting that changes in reported perceived school safety lead to decreases in educational attainment, not the other way around. This study also found that feeling unsafe at school had statistically significant effect on educational attainment as students are exposed to greater in-school violence and disruption [40]. In general, security measures, such as metal detectors digital surveillance technology, were related to lower perceptions of safety [47,48,49,50]. An exception was having a security officer at the school, which was associated with higher perceptions of safety [43,48]. Fair, clear, and consistent school rule enforcement was associated with a greater feeling of safety [31,38,50]. Mooij and Fettelaar [42] reported that students who went to larger schools were more likely to report feeling safe. Perumean-Chaney and Sutton [50] found that students who were in larger classes were less likely to feel safe than those in smaller classes. Students with a higher perception of their school environment were more likely to report feeling safer at school [44,47]. Within school climate items, school connection was the largest single contribution to the overall prediction of a sense of safety at school [47].

Another factor that shaped how safe students felt about safety was their relationship with others. Students who felt that their teachers care about them [38,44] and had high levels of trust in them [65] were more likely to feel safe at school. When teachers were seen to be unfair, this was reflected in lower perceptions of safety [66]. Students who reported that family cohesion was important to them, and who came from intact families, were more likely to feel safe at school [39,42]. Students who talked to their families more about activities and events at school were more likely to report better perceptions of school safety [38]. Having close friends [39] and feeling that making friends was easy [38] were associated with a higher sense of school safety.

The use, and availability, of substances and weapons were also associated with how safe students felt. Five reported that significantly lower perceived school safety if substances were available and used at school [31,39,44,45,55]. The most common types of substances were drugs, followed by alcohol and cigarettes. Students who carried a weapon at school were more likely to feel that their school was unsafe [36,62], as did students who saw others carrying weapons at school [38,44].

#### 3.3.2. Interrelations between Surrounding Environments

The interrelations of the different surrounding environments include interactions between family members and schoolteachers, which has rarely been studied. Hong and Eamon [38] found that parents getting involved by attending school meetings and events was not significantly associated with perceived school safety. Other variables, such as relationships with friends and family members and social workers being involved with the family, have not been explored.

#### 3.3.3. Environments That Students Did Not Actively Participate in

The environments that school students did not actively participate in included social structures that influenced the child’s immediate settings, such as their local community [67]. This has not been widely studied in the included studies. Students who felt their neighborhood was safer were more likely to feel safe at school [38,47,50]. This result suggested that other factors that students were not involved in, such as racism and discrimination and employment opportunities could also have been associated with perceived school safety [19,20].

We did not find any cultural context and life transitions factors that were associated with school safety or sociohistorical events, such as economic recessions, anti-discrimination laws, changing schools and puberty.

**Figure 2:**
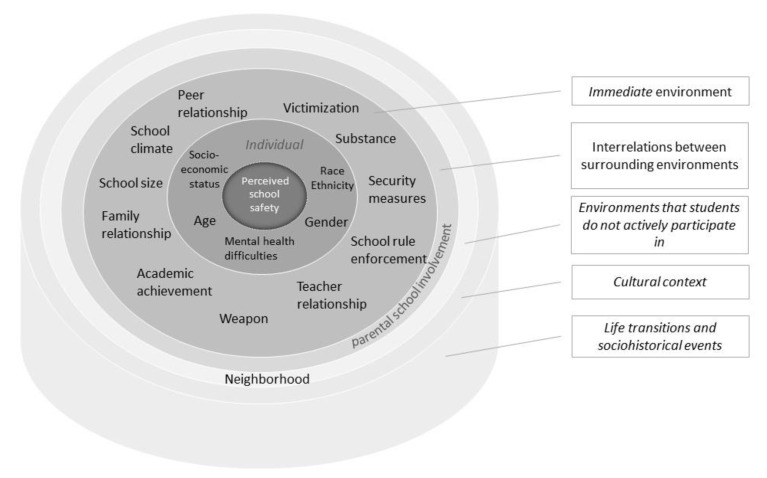
An adapted ecological systems model of the multiple domains related to perceived school safety.

Table 3 presents a summary of the factors that were associated with a sense of school safety, and Figure 2 illustrates an adapted ecological systems model of the multiple domains in relation to perceived school safety among children and adolescents.

**Table 3:** Summary of factors and mental health difficulties associated with school safety.

Factors	Studies, *n*	Factors	Relation	Studies
Sex	18	Being a boy	−	[31,34,38,45,46,47]
		Being a girl	−	[39,42,48,49,50]
		Sexual minority	−	[22,33,34,51]
		No association		[32,35,68]
Race/Ethnicity	11	Non-white	−	[34,35,39,43,45,48,49,50]
		American versus Chinese	−	[52]
		Arab versus Jewish	−	[47]
		No association		[31]
Age	7	Older age	−	[38,49,52,53]
		Younger age	−	[42,47,48]
Socioeconomic status	2	Low socioeconomic status	−	[38,49]
Mental health difficulties	10	Depression	−	[45,54,55,56]
		Suicidal ideation and attempts	−	[55,56,57,58,59]
		Self-harm	−	[32,59]
		Posttraumatic stress disorder	−	[60]
		Posttraumatic growth	−	[60]
		Mental health problems (SDQ)	−	[61]
Victimization	20	Bullying	−	[31,35,45,49,53,62,63,66,68,69,70]
		Youth violence	−	[31,39,44,49,50,70]
		Emotional or psychological violence and witnessing violence	−	[45,47,49,56,70,71]
		Intimate partner violence	−	[37]
		Sexual violence	−	[57]
		Teacher-to-student victimization	−	[72]
Academic achievement	5	Low academic achievement	−	[40,42,50,55,64]
Security measures	5	Security measures use	−	[47,48,49,50]
		Security officer present	+	[43,48]
School rule enforcement	4	Communicated, fair rules	+	[31,38,50]
		No association	−	[42]
School size	2	Larger school size	+	[42]
		Larger class size		[50]
School climate	2	Better school climate	+	[44,47]
Teacher relationship	4	Teacher care	+	[38,44]
		Trust in teacher	+	[65]
		Teacher unfair	−	[66]
Family relationship	3	Family cohesion and intactness	+	[39,42]
		Discussing school activities with parents	+	[38]
Peer relationship	2	Having close friends	+	[39]
		Making friends easily	+	[38]
Substance	5	Drugs	−	[31,39,44]
		Alcohol	−	[44,45,55]
		Cigarettes	−	[38,55]
Weapon	4	Carrying weapons	−	[36,62]
		Seeing weapons carried	−	[38,44]
Parental school involvement	1	No association		[38]
Neighborhood	3	Safe neighborhood environment	+	[42,47,50]
Truant	1	Playing truant	−	[31]
Weight-related health behaviors	1	More physical activity	+	[51]
		More participation in physical education	+	[51]
			−	[51]
		Unhealthy eating habits	−	[42]
Delinquency	1	Higher rates of expulsion and suspension	−	[41]
Sleeping	1	Insufficient sleep	+	[42]
Religion	1	Being baptized	+	[46]
Sexual debut	1	Older sexual debut	−	[39]
Curricular differentiation	1	School’s curricular differentiation	+	[39,42]
Language proficiency	1	Lack of English proficiency	−	[65]
Student identification	1	Student identifies with school	+	[42]
Feeling home	1	Not feeling home in the country	−	[42]

Note: (−) = associated with feeling unsafe, (+) = associated with feeling safe.

### 3.4. Cross-Cultural Studies

One cross-cultural study that compared China and the United States met our criteria and passed the quality assessment [52]. It found that Chinese students had significantly higher perceptions of school safety than American students in middle school and high school, but there were no significant differences in elementary school.

## 4. Discussion

This study had four key findings. First, a remarkable number of children and adolescents did not feel safe at school. Second, students’ perceptions of school safety were associated with factors such as being bullied. Third, not feeling safe at school was related to mental health difficulties, such as depressive symptoms and suicidal behavior. Fourth, there was only one cross-cultural study on perceived school safety met our criteria, and only four studies were carried out in non-western societies.

The mean prevalence of students who felt unsafe was 19.4%, and it was alarmingly high in some studies. This high prevalence highlights the need of strong political leadership, a robust legal and policy framework to promoting a safe learning environment, including the use of positive discipline [73]. There were wide variations between countries, from 6.1% in a study from the United States [37] to 69.1% in a study from Japan [32]. The large variation in the results was influenced by differences in the study methods, the lack of a definition for perceived school safety and how it was measured. The findings were mainly based on studies carried out in single western countries, which made it impossible to identify culture-specific differences in perceived school safety.

The analysis revealed that multiple factors affected how safe children and adolescents felt at school. The literature consistently reported strong associations between being bullied, youth violence and low levels of school safety, which is understandable, as victims may feel scared to go to school and avoid going [74,75,76]. Students expect teachers to keep them safe and actively address bullying [77,78]. However, teachers do not always successfully intervene, because they may be unaware of bullying [79,80] or may even feel it is unnecessary to intervene [81].

There were mixed results regarding sex. Some studies said that boys were more likely to feel unsafe at school than girls [31,34,38,45,46,47], due to their higher involvement in disruptive activities and bullying [82,83]. However, some studies said girls had greater fears than boys [39,42,48,49,50], and other studies found no significant differences between sexes [32,35,68]. These findings suggest that a sense of feeling unsafe at school is a shared issue for both boys and girls.

The studies reported relatively persistent results that sexual minority youths and non-white students felt unsafe at school. This might be because sexual minority youths [84,85] and students from ethnic minorities [86,87] faced a higher risk of being bullied. We were surprised that several studies reported that older students had lower perceived school safety, as there is generally a decrease in bullying as children grow older [88,89]. It is possible that other factors associated with lower perceived school safety could explain these findings, such as the emotional problems that are more prevalent among adolescents than younger children [90].

Feeling unsafe at school was associated with various mental health problems, including emotional problems and suicidal behavior. Previous research has shown that mental health problems were associated with being bullied [91,92,93,94], and others have reported a bi-directional association in which mental health problems both preceded and followed being bullied [95]. Because being bullied was also associated with perceived school safety, the associations between mental health, victimization and perceived school safety appear complex. Understanding and addressing these associations could help to tackle absenteeism, because as many as 10–14% of students avoided school because they felt unsafe [96,97]. Mental health problems [98,99] and being bullied [74,89] have also been associated with absenteeism. The associations between feeling safe at school, mental health difficulties, and victimization and absenteeism emphasize the importance of comprehensive interventions for the whole school community, targeted sub-groups who are at high risk, and students who have already developed symptoms. These should be provided according to the nature and intensity of the students’ needs [10].

Strengthening school security measures has become a common preventive response to school violence [100]. However, using security measures, such as metal detectors and digital surveillance technology, had a negative impact on students’ perceptions of school safety. The only exception was a school security officer at school, which was associated with higher perceived school safety [43,48]. It is possible that metal detectors inconvenience students [101] and that they do not like feeling of being searched and monitored by security devices [48,102]. This could have a negative impact on their sense of safety by, for example, reminding them of a potential threat. On the one hand, risky behaviors, such as school violence and substance use, are more likely when there are no consistent, clear rules for students [103]. On the other hand, when fair, clear, and consistent school rules were enforced, this was associated with a higher sense of school safety. Trusting teachers and feeling that they cared for students were also associated with higher perceptions of school safety. Smaller classes enabled students to have more individual attention from, and active interaction with, teachers [104]. A longitudinal study showed a positive long-term impact of smaller classes on students, including higher academic achievements and completing education [105].

It appears that complex interactions of social, psychological, and biological factors shaped students’ emotional sense of safety and that these factors moderated the effects that each other had on how safe children and adolescents felt at school. Although perceived school safety was measured with a single question in most studies, it was a strong independent risk factor that was associated with being bullied and mental health difficulties. When students experience a combination of these factors associated with poor school safety, it may put them at greater risk for behaviors and mental and behavioral health concerns. This finding suggests that these most vulnerable students may need additional targeted actions by, for example, providing selective prevention interventions.

Only one cross-cultural study on perceived school safety fulfilled the inclusion criteria. Bear et al. [52] reported that Chinese students had significantly higher perceptions of school safety than American students. A Master’s degree thesis by Gong [106] was not included, as it did not pass the quality assessment, but that also showed that Chinese students’ perceptions of school safety were significantly higher than American students, across ages and sexes [106]. The differences in the prevalence of school safety among countries might be explained by different cultural values and norms, such as self-perfection, respecting teachers, and social harmony [52,106]. Given increasing international immigration [107] and the fact that ethnicity and feeling at home in the country is associated with feeling safe, future studies should examine how safe immigrant children feel at school compared to their native-born peers. This was the first review to provide a comprehensive overview of studies on how safe children and adolescents felt at school and associated mental health difficulties. Based on our findings, future studies should use valid measurements and provide clear information about the scoring and interpretation of scales. They also need to provide clear definitions of perceived school safety to improve the consistency of reporting. In future reviews, it would be important to include studies not published in English to gain a wider understanding of school safety. Most of the factors that were associated with perceived school safety were in the student’s immediate environment and attention to other settings was rather limited. It would be interesting for future studies to examine associations between perceived school safety and factors in other settings, such as the interrelations of different surrounding environments, environments that the child does not actively participate in, cultural contexts, life transitions, and sociohistorical events. Most of the studies we reviewed were published between 2016–2020, indicating an increasing research interest in school safety. This might have been influenced by the increased number of school shooting in the United States in recent years [108]. Cross-cultural perspectives should be considered a key aspect of future investigations into perceptions of school safety, including non-western countries. Ecological perspectives would also enhance our understanding of how different associated factors interact and moderate each other’s effects to recognize and support the most vulnerable students.

Our review had several limitations, including the fact that we only studied papers published in English. This may explain why there were significantly fewer studies from non-western countries. All of the studies, except one, had cross-sectional designs that were insufficient to establish causal relationships. Inconsistencies in the measurement tools used in the studies and the lack of a clear definition of school safety limited our ability to synthesize the results. A strength was that different guidelines were used to ensure a transparent and complete reporting process, as well as the quality of evidence in the review. Despite these limitations, this review provides unique findings on the overall prevalence of perceived school safety and the various factors and mental health difficulties associated with that.

## 5. Conclusions

Global educators and policymakers need to be aware that a high prevalence of students feel unsafe at school. The findings of our review highlight the importance of various social, psychological, and biological factors that contributed to low perceived school safety, especially being bullied. In addition, our findings suggest that teachers, family members, and friends play a key role in making students feel safe at school. Longitudinal studies are needed to examine the mechanisms of school safety. Possible adversities due to a low sense of safety should be a key focus of future investigations. Studies also need to focus on cross-cultural perspectives and include non-western countries.

## Figures and Tables

**Table 1:** Description of the 43 studies included in the review.

		Number of Studies	% of Included Studies
Methodology	Cross sectional	40	93
	Longitudinal	3	7
Finding	Prevalence	17	40
	Factors	42	98
	Mental health	10	23
	Cross-cultural study	1	2
Location	North America	34	79
	South America	1	2
	Europe	4	9
	Asia	3	7
	United States and China	1	2
Setting	Elementary school	1	2
	Secondary school	38	88
	Elementary and secondary school	4	9
Publication year	2004–2016	13	30
	2016–2020	30	70
Informants	Only self-reports	43	100
Measure	Single item	25	58
	Multiple items	18	42
Response	4-point Likert scale	23	53
	5-point Likert scale	16	37
	3-point Likert scale	1	2
Methodology	Dichotomous answer categories	4	9

## Data Availability

No new data were created in this study. Data sharing is not applicable to this article.

## Supplementary Materials

The following are available online at https://www.mdpi.com/2227-9067/8/3/232/s1. Table S1: Search strings used in databases. Table S2: Characteristics of the studies that were included in this review. Table S3: Results of quality assessment of the observational included studies.
