# Supplementary material for: Feeling Unsafe at School and Associated Mental Health Difficulties among Children and Adolescents: A Systematic Review
Source: Children (Basel). 2021 Mar 17;8(3):232. doi: 10.3390/children8030232 (PMC8002666; doi:10.3390/children8030232)
Supplement: Supplementary file 1 [file children-08-00232-s001.pdf]

Feeling Unsafe at School and Associated Mental Health Difficulties among Children and Adolescents: A Systematic Review - Supplementary Materials -

Table S1. Search strings used in databases.

Databases	Search String
PubMed	"school safet*" AND (child* OR adolesc* OR teen* OR preteen* OR youth* OR "Child"[Mesh] OR "Adolescent"[Mesh])
CINAHL (EBSCO)	"school safet*" AND (child* OR adolesc* OR teen* OR preteen* OR youth* OR MH "Child" OR MH "Adolescence")
PsycINFO (EBSCO)	("school safet*" OR DE "school safety") AND (child* OR adolesc* OR teen* OR preteen* OR youth*)
ERIC (EBSCO)	("school safet*" OR DE "School Safety") AND (child* OR adolesc* OR teen* OR preteen* OR youth* OR DE "Youth" OR DE "Adolescents")
Web of Science	"school safet*" AND (child* OR adolesc* OR teen* OR preteen* OR youth*)

Table S2. Characteristics of the studies that were included in this review.

Author	Country	Participants	Response rate (%)	Age (Grade)	Measure	Response	Data collected year	Design	Setting
Arora 2018	US	1,354	82	M 14.20 (1993) 17.84 (1995)	8 items (e.g., I do not feel safe)	3, 4-point Likert	1993, 1995	CS	S
Atteberry-Ash 2019	US	11,986	85	M 15.7	I feel safe at my school	4-point Likert	2015	CS	S
Bachman 2011	US	20,138	82	5,8,11 grades	I feel safe in my school	Yes/No 5-point Likert	2007	CS	E/S
Bear 2018	US, China	7,261	81.9 (US) 94.9 (China)	3–5, 7–8, 10–12 grades	3 items (e.g., Students are safe in the hallways)	4-point Likert	NR	CS	E/S
Bowser 2018	US	5,138	96	6-8 grades	How often do you feel safe and secure at school?	5-point Likert	2015	CS	S
Docherty 2020	US	122,840	62-85	8,10,12 grades	I feel safe at my school	4-point Likert	2014,2016,2018	CS	S
Earnest 2016	US	75,590	90	9,12 grades	I feel safe at school	4-point Likert	2010	CS	S
Eisenberg 2007	US	83,731	97	6,9,12 grades	3 items (e.g., I feel safe at school)	4-point Likert	2004	CS	S
Esselmont 2014	US	7,464	82	6-10 grades	I feel safe at this school	5-point Likert	2002	CS	S
Eugene 2020	US	9,518	NR	10, 12 grades	3 items (e.g., Does not feel safe at this school)	4-point Likert	2002, 2004	LS	S
Garnett 2018	US	2,481	95	M 14.5	4 items (e.g., I feel safe at school)	5-point Likert	2015	CS	S
Gase 2017	US	33,572	NR	6-12 grades	2 items (e.g., student feels safe at school)	5-point Likert	2015	CS	S

Gini 2018	Italy	1,378	85	6-10 grades	I feel safe in my class	5-point Likert	2016	CS	S
Glew 2008	US	5,391	87	7,9,11 grades	I feel safe at my school	5-point Likert	2002	CS	S
Goldweber 2013	US	12,763	100	6-12 grades	2 items (e.g., I feel safe at this school)	4-point Likert	2008	CS	S
Hamada 2018	Japan	1,865	92.8	M 13.9	Do you feel secure at school?	4-point Likert	2011	CS	S
Hong 2012	US	1,249	70	10-15 years old	I don't feel safe at this school	4-point Likert	2004, 2006	CS	S
Hong 2016	US	4,118	94	9-12 grades	I don't feel safe at this school	4-point Likert	1995	CS	S
Konishi 2017	Canada	48,874	97	8-12 grades	3 items (e.g., I feel safe at school)	5-point Likert	2008	CS	S
Lacoe 2020	US	658,122	80	6-8 grades	I am safe in my classes	4-point Likert	2007-2010	LS	S
Lear 2020	US	13,449	66	6,8,10,12 grades	How often do you feel unsafe at school	5-point Likert	2016	CS	S
Lindstrom 2018	US	54,350	NR	6-12 grades	4 items (e.g., I feel safe at this school)	4-point Likert	2014, 2016	CS	S
López 2020	Chile	50,344	50<	5-8 grades	2 items (e.g., I feel fear in my school)	4-point Likert	2017	CS	E
Lorenzo 2016	US	1,919	92	9-11 grades	6 items (e.g., My school is a safe and protected place)	4-point Likert	2005, 2006, 2007	LS	S
March, 2010	US	6,989	83	9-12 grades	Several items (e.g., felt unsafe at school or on way to or from school)	NR	2003	CS	S
Meldrum 2018	US	7,958	69	M 14.36	I feel safe at my school	4-point Likert	2017	CS	S

Mitchell 2018	US	5,441	66	3-12 grades	9 items (e.g., I feel safe inside the school)	5-point Likert	2009	CS	E/S
Mooij, Fettelaar 2012	Netherlands	71,560	NR	M 14.3	7 items different locations	Yes/No	2008	CS	S
Moore 2018	US	1,169	NR	9,11 grades	2 items (e.g., I feel safe in my school)	5-point Likert	2013	CS	S
Mowen 2019	US	15,362	NR	10 grade	2 items (e.g., if they felt safe at the school)	4-point Likert	2002	CS	S
Nijs 2014	Netherlands	11,130	98	11-19 years old	Do you ever feel unsafe at school?	4-point Likert	2007	CS	S
Pampati 2020	US	542	66.8	13< (88%= >15)	Do you feel safe at school?	Yes/No	2016	CS	S
Pentek 2018	US	126,868	NR	8,9,11 grades	I feel safe at school	4-point Likert	2016	CS	S
Perumean-Chaney 2013	US	13,386	NR	7-12 grades	You feel safe in your school	4-point Likert	1995, 1996	CS	S
Pistella 2020	US	31,609	NR	6-12 grades	2 items (e.g., I feel safe in my school)	5-point Likert	2013-2015	CS	S
Radu 2018	US	4,130	90	12-14 years old	Do you feel safe at school?	4-point Likert	1997	CS	S
Rose 2018	US	9,619	67	9-12 grades	Do you feel safe at your school?	Yes/No	2016	CS	S
Skiba 2004	US	2,231	59-87	6-12 grades	Overall, I feel that this school is a safe school.	5-point Likert	2001	CS	S
Taliaferro 2019	US	1,635	75	9,11 grades	I feel safe at school	4-point Likert	2016	CS	S
Tiiri 2020	Finland	3,997	90.2 (2008) 91.8 (2014)	M 14.4 7,9 grades	I feel safe at school	4-point Likert	2008, 2014	CS	S
Vaillancourt 2010	Canada	11,152	94	4-12 grades	Several items different locations and time	5-point Likert	2008	CS	E/S

Yablon 2010	Israel	2,199	NR	6,8,10 grades	Several items different locations	5-point Likert	NR	CS	S
Yablon 2019	Israel	609	94	9-12 grades	6 items (e.g., I do not feel safe in this school)	5-point Likert	NR	CS	S

Note: Schools: elementary (E) and secondary (S). CS = Cross-sectional, LS = Longitudinal, NR = Not reported, M = mean age.

Table S3. Results of quality assessment of the observational included studies.

Author	CK1	CK2	CK3	CK4	CK5	CK6	CK7	CK8	CK9	CK10	CK11	CK12	CK13	CK14	Overall quality
Arora 2018	Yes	Yes	Yes	Yes	Yes	No	No	NA	Yes	No	No	NA	NA	Yes	Good
Atteberry-Ash 2019	Yes	Yes	Yes	Yes	Yes	No	No	NA	Yes	No	No	NA	NA	Yes	Fair
Bachman 2011	No	Yes	Yes	Yes	Yes	No	No	NA	No	No	No	NA	NA	Yes	Fair
Bear 2018	Yes	Yes	Yes	Yes	Yes	No	No	NA	Yes	No	No	NA	NA	Yes	Good
Bowser 2018	No	Yes	Yes	Yes	Yes	No	No	Yes	No	No	No	NA	NA	Yes	Fair
Docherty 2020	Yes	Yes	Yes	Yes	Yes	No	No	Yes	Yes	No	Yes	NA	NA	Yes	Fair
Earnest 2016	Yes	Yes	Yes	Yes	Yes	No	No	NA	Yes	No	No	NA	NA	Yes	Good
Eisenberg 2007	No	Yes	Yes	Yes	Yes	No	No	NA	Yes	No	No	NA	NA	Yes	Fair
Esselmont 2014	Yes	Yes	Yes	Yes	Yes	No	No	NA	Yes	No	No	NA	NA	Yes	Good
Eugene 2020	Yes	Yes	Yes	Yes	Yes	Yes	Yes	Yes	Yes	No	Yes	NA	Yes	Yes	Good
Garnett 2018	Yes	Yes	Yes	Yes	Yes	No	No	NA	Yes	No	No	NA	NA	Yes	Good
Gase 2017	Yes	Yes	Yes	Yes	Yes	No	No	NA	No	No	No	NA	NA	Yes	Fair
Gini 2018	Yes	Yes	Yes	Yes	Yes	No	No	NA	Yes	No	No	NA	NA	No	Fair
Glew 2008	Yes	Yes	Yes	Yes	Yes	No	No	NA	Yes	No	No	NA	NA	Yes	Good
Goldweber 2013	Yes	Yes	Yes	Yes	Yes	No	No	NA	Yes	No	No	NA	NA	Yes	Good
Hamada 2018	Yes	Yes	Yes	Yes	Yes	No	No	NA	Yes	No	No	NA	NA	Yes	Fair
Hong 2012	No	Yes	Yes	Yes	Yes	No	No	NA	Yes	No	No	NA	NA	No	Fair
Hong 2016	Yes	Yes	Yes	Yes	Yes	No	No	NA	Yes	No	No	NA	NA	No	Good
Konishi 2017	Yes	Yes	Yes	Yes	Yes	No	No	NA	Yes	No	No	NA	NA	Yes	Good
Lacoe 2020	Yes	Yes	Yes	Yes	Yes	Yes	Yes	Yes	Yes	Yes	Yes	NA	Yes	Yes	Good
Lear 2020	Yes	Yes	Yes	Yes	Yes	No	No	Yes	Yes	No	Yes	NA	NA	Yes	Good
Lindstrom 2018	Yes	Yes	NR	Yes	Yes	No	No	NA	Yes	No	No	NA	NA	Yes	Fair
López 2020	Yes	Yes	Yes	Yes	Yes	No	No	Yes	Yes	No	Yes	NA	NA	Yes	Good
Lorenzo 2016	Yes	Yes	Yes	Yes	Yes	No	No	NA	Yes	Yes	No	NA	Yes	Yes	Good
March, 2010	Yes	Yes	Yes	Yes	Yes	No	No	NA	Yes	No	No	NA	NA	No	Fair
Meldrum 2018	Yes	Yes	Yes	Yes	Yes	No	No	NA	No	No	No	NA	NA	Yes	Fair
Mitchell 2018	Yes	Yes	Yes	Yes	Yes	No	No	NA	Yes	No	No	NA	NA	Yes	Good
Mooij, Fettelaar 2012	Yes	Yes	NR	Yes	Yes	No	No	NA	Yes	No	No	NA	NA	Yes	Fair

Moore 2018	Yes	Yes	NR	Yes	Yes	No	No	NA	Yes	No	No	NA	NA	Yes	Fair
Mowen 2019	Yes	Yes	NR	Yes	Yes	No	No	NA	Yes	No	No	NA	NA	Yes	Fair
Nijs 2014	Yes	Yes	Yes	Yes	Yes	No	No	NA	Yes	No	No	NA	NA	Yes	Good
Pampati 2020	Yes	Yes	Yes	Yes	Yes	No	No	Yes	Yes	No	Yes	NA	NA	Yes	Fair
Pentek 2018	Yes	Yes	NR	Yes	Yes	No	No	NA	Yes	No	No	NA	NA	Yes	Good
Perumean-Chaney 2013	Yes	Yes	Yes	Yes	Yes	No	No	NA	Yes	No	No	NA	NA	Yes	Good
Pistella 2020	Yes	Yes	NR	Yes	Yes	No	No	Yes	Yes	No	Yes	NA	NA	Yes	Fair
Radu 2018	Yes	Yes	Yes	Yes	Yes	No	No	NA	Yes	No	No	NA	NA	Yes	Good
Rose 2018	Yes	Yes	Yes	Yes	Yes	No	No	NA	Yes	No	No	NA	NA	Yes	Good
Skiba 2004	No	Yes	Yes	Yes	Yes	No	No	NA	Yes	No	No	NA	NA	Yes	Fair
Taliaferro 2019	Yes	Yes	Yes	Yes	Yes	No	No	NA	Yes	No	No	NA	NA	Yes	Good
Tiiri 2020	Yes	Yes	Yes	Yes	Yes	No	No	NA	Yes	No	No	NA	NA	Yes	Good
Vaillancourt 2010	No	Yes	Yes	Yes	Yes	No	No	NA	Yes	No	No	NA	NA	Yes	Fair
Yablon 2010	Yes	Yes	Yes	Yes	Yes	No	No	NA	Yes	No	No	NA	NA	Yes	Good
Yablon 2019	Yes	Yes	Yes	Yes	No	No	No	NA	Yes	No	No	NA	NA	Yes	Fair

Note: Quality of included studies was assessed using the National Institutes of Health (NIH) Quality Assessment tool for Observational Cohort and Cross-Sectional Studies (https://www.nhlbi.nih.gov/health-pro/guidelines/in-develop/cardiovascular-risk-reduction/tools/cohort). CK 1. Was the research question or objective in this paper clearly stated? CK 2. Was the study population clearly specified and defined? CK 3. Was the participation rate of eligible persons at least 50%? CK 4. Were all the subjects selected or recruited from the same or similar populations (including the same time period)? Were inclusion and exclusion criteria for being in the study prespecified and applied uniformly to all participants? CK 5. Was a sample size justification, power description, or variance and effect estimates provided? CK 6. For the analyses in this paper, were the exposure(s) of interest measured prior to the outcome(s) being measured? CK 7. Was the timeframe sufficient so that one could reasonably expect to see an association between exposure and outcome if it existed? CK 8. For exposures that can vary in amount or level, did the study examine different levels of the exposure as related to the outcome (e.g., categories of exposure, or exposure measured as continuous variable)? CK 9. Were the exposure measures (independent variables) clearly defined, valid, reliable, and implemented consistently across all study participants? CK 10. Was the exposure(s) assessed more than once over time? CK 11. Were the outcome measures (dependent variables) clearly defined, valid, reliable, and implemented consistently across all study participants? CK 12. Were the outcome assessors blinded to the exposure status of participants? CK 13. Was loss to follow-up after baseline 20% or less? CK 14. Were key potential confounding variables measured and adjusted statistically for their impact on the relationship between exposure(s) and outcome(s)? CK, check list; CD, cannot be determined; NA, not applicable; NR, not reported.
